# CHA_2_DS_2_-VASc Score in Predicting Visual Acuity Outcomes Following Retinal Vein Occlusion

**DOI:** 10.1155/2024/3054783

**Published:** 2024-10-21

**Authors:** Achia Nemet, Raimo Tuuminen, Tzadok Yona, Gilad Plopsky, Michal Katz, Natali Glinkin, Olga Lelchuk, Joseph Pikkel

**Affiliations:** ^1^Department of Ophthalmology, Assuta Ashdod University Medical Center, Ashdod, Israel; ^2^Ben-Gurion University of the Negev, Beer Sheva, Israel; ^3^Helsinki Retina Research Group, Faculty of Medicine, University of Helsinki, Helsinki, Finland; ^4^Department of Ophthalmology, Kymenlaakso Central Hospital, Kotka, Finland; ^5^Faculty of Health Sciences, Ben-Gurion University of the Negev, Beer Sheva, Israel; ^6^Sackler School of Medicine, Tel-Aviv University, Tel-Aviv, Israel

**Keywords:** CHA_2_DS_2_-VASc score, retinal vein occlusion, visual acuity

## Abstract

**Purpose:** To find whether the CHA_2_DS_2_-VASc score, a system for stratifying stroke risk among patients with atrial fibrillation, correlates with visual acuity prognosis following retinal vein occlusion (RVO).

**Participants and Methods:** This retrospective study included 83 eyes of 83 patients with a diagnosis of branch or central RVO between June 2017 and August 2022 with at least 12 months of follow-up in Assuta Ashdod Medical Center, Ashdod, Israel. The patients were divided into three groups, with CHA_2_DS_2_-VASc scores of 0–2 (*N* = 31), 3–5 (*N* = 45), or 6–9 (*N* = 7). The change in best-corrected visual acuity (BCVA) between the groups was examined about 1 year after the presentation of RVO.

**Results:** The patient mean age was 67.9 ± 13.8 years; 38.6% were women. The mean visual acuity was 0.83 ± 0.67 LogMAR units at the first admission and 0.78 ± 0.80 LogMAR units at the last visit. Patients with a CHA_2_DS_2_-VASc score from 6 to 9 had a significantly poorer BCVA prognosis at 1-year (+0.60 ± 0.94 [−0.27, 1.47] LogMAR units) compared to groups with a CHA_2_DS_2_-VASc score from 3 to 5 (−0.01 ± 0.65 [−0.20, 0.19] LogMAR units) and a CHA_2_DS_2_-VASc score from 0 to 2 (−0.12 ± 0.59 [−0.33, 0.10] LogMAR units) (*p* = 0.038).

**Conclusions:** Following RVO, patients with a CHA_2_DS_2_-VASc score of 6 or higher had a worse prognosis in their visual acuity than patients with a lower score.

**Summary**

• Retinal vein occlusion (RVO) is a common vascular disorder leading to a reduction in visual acuity.
• The CHA_2_DS_2_-VASc score is used for stroke risk stratification in individuals with atrial fibrillation.
• CHA_2_DS_2_-VASc score of ≥6 was associated with impaired visual acuity outcomes after RVO.

## 1. Introduction

Retinal vein occlusion (RVO) is the second most common cause of vision deterioration from retinal vascular disease, following diabetic retinopathy [1, 2]. It is characterized by venous stasis, retinal ischemia, and cystoid macular edema (CME). RVO can be classified into two main types, branch (BRVO) and central (CRVO) forms of the disease. Systemic risk factors associated with RVO include hypertension, diabetes mellitus, hyperlipidemia, and atherosclerosis [3, 4].

Treatment options for RVO include intravitreal injections of antivascular endothelial growth factors (VEGFs), corticosteroids, or retinal laser photocoagulation. Additionally, systemic control of risk factors and close monitoring for the development of ocular comorbidities such as neovascular glaucoma are essential [5–7].

Close collaboration between ophthalmologists and other healthcare professionals is crucial for managing RVO effectively [8]. The CHA_2_DS_2_-VASc score is a system used to stratify the risk of stroke in atrial fibrillation patients [9, 10]. The score considers various risk factors such as congestive heart failure, hypertension, age and gender, diabetes mellitus, and prior stroke or transient ischemic attack (TIA). Each risk factor is assigned a score, and the total score helps in determining the appropriate level of anticoagulation therapy for stroke prevention [11].

Although RVO and the CHA_2_DS_2_-VASc score may appear unrelated, they represent the complex relationship between the cardiovascular system and the eye health. Both conditions emphasize the need for a comprehensive approach to medical care, where physicians consider various risk factors and clinical tools to accurately diagnose and manage patients. By understanding the underlying mechanisms and risk factors associated with RVO and utilizing scoring systems like the CHA_2_DS_2_-VASc score, healthcare professionals can provide optimal care and improve patient outcomes.

Here we examined the association between the CHA_2_DS_2_-VASC score and the visual acuity prognosis at 1 year from the presentation of RVO.

## 2. Materials and Methods

Patients with RVO and CME were followed for 1 year. All participants received intravitreal anti-VEGF treatment for the treatment of the disease. Baseline and the 1-year BCVA were analyzed from every patient in relation to their CHA_2_DS_2_-VASC score. All data for this study were collected and analyzed in accordance with the policies and procedures of the Institutional Review Board, Assuta Ashdod Medical Center, Ashdod, Israel, and the tenets set forth in the Declaration of Helsinki.

### 2.1. Study Participants and CHA_2_DS_2_-VASC Score Groups

This retrospective study included patients who visited the ophthalmology clinic or ophthalmology emergency room at Assuta Ashdod Medical Center with a diagnosis of RVO (branch [*N* = 24] or central [*N* = 59]) between June 2017 and August 2022. These were analyzed together based on similar ophthalmic treatment protocol. Inclusion criteria were age more than 18 years, intravitreal anti-VEGF treatment, a minimum of 1 year follow-up and no history of other retinal comorbidities. All patients who had undergone any ocular procedure other than phacoemulsification and intraocular lens implantation prior to RVO (e.g., intravitreal injections, retinal laser photocoagulation, and intraocular surgeries) were excluded from the study (Supporting Figure 1).

CHA_2_DS_2_-VASc score groups were categorized as 0–2, three to five, and six to nine, based on the literature associated with a prognostic value of the scores for post-discharge outcomes, risks for adverse outcomes and prediction of death in elderly patients: low, moderate to high, and very high risk, respectively [12–14].

### 2.2. Clinical Examination and Treatment

After RVO, all patients received anti-VEGF injections according to the *pro re nata* (PRN; as needed) protocol after a periodic optical coherence tomography (OCT) examination and with the clinical decision of a retinal specialist.

### 2.3. Statistical Analysis

Continuous data are expressed as mean ± standard deviation (SD) and categorical data as frequency counts (percentages). Levene's test of homogeneity of variance was used for the Student's *T*-test.

Normality of the data was assessed visually using density plots. Differences between groups were determined using the one-way ANOVA test. The Tukey's Honest Significant Difference (HSD) test was used as a post hoc test following the ANOVA test. The significance level was set to *p* < 0.05. All analyses were performed with R Software (Version 4.3.0).

## 3. Results

A total of 83 eyes of 83 patients with a mean age of 67.9 ± 13.8 years were included in this study; 38.6% were females (The data collection can be seen in the attached flowchart).

The mean BCVA at the first admission was 0.81 ± 0.67 LogMAR units and the mean intraocular pressure (IOP) was 16.4 ± 6.7 mmHg (8.4% had IOP > 21 mmHg). At 1 year, the mean BCVA was 0.78 ± 0.80 LogMAR units and the mean IOP was 17.0 ± 8.4 mmHg (7.2% had IOP > 21 mmHg). The CHA_2_DS_2_-VASc score distribution is shown in Table 1.

The mean foveal thickness and central subfield macular thickness at the first admission were 383.3 ± 84.9 *μ*m and 470.3 ± 197.3 *μ*m, respectively. The mean macular volume at the first admission was 10.5 ± 2.4 *μ*m^3^.

Next, we divided the patients into 3 groups: patients with a CHA_2_DS_2_-VASc score from 0 to 2, from 3 to 5 and from 6 to 9.  Table 2 presents the distribution of the 83 patients included in this study.

At admission, the mean BCVA was 0.64 ± 0.56 LogMAR units for patients with the CHA_2_DS_2_-VASc score of 0–2, 0.86 ± 0.67 LogMAR units for those with the score of three to five and 1.24 ± 0.89 LogMAR units for those with the score of 6–9. At 1-year, the respective values were 0.52 ± 0.60, 0.85 ± 0.85 and 1.84 ± 1.43 LogMAR units, respectively. The changes in BCVA at 1-year compared to baseline were −0.12 [−0.33, 0.10], −0.01 [−0.20, 0.19] and +0.60 [−0.27, 1.47] LogMAR units, respectively (95% CI, *p*=0.038, Table 2, Bar 1 and Density Plot (Supporting Information available (here))).

## 4. Discussion

The CHA_2_DS_2_-VASc score is a widely used clinical tool for predicting the risk of ischemic events in patients with atrial fibrillation (AF) [12]. Ischemic events, including strokes and systemic embolism, are common complications of AF and are associated with significant morbidity and mortality [13]. The CHA_2_DS_2_-VASc score incorporates various clinical risk factors, including congestive heart failure, hypertension, age, diabetes mellitus, prior stroke or TIA, vascular disease, and female sex [14, 15]. Currently, no scientific evidence exists in regard to the relationship between CHA_2_DS_2_-VASc score and visual acuity after RVO, although occlusions are commonly associated with various factors including hypertension, diabetes, hyperlipidemia, and other systemic diseases. Apparently, both the CHA_2_DS_2_-VASc score alike RVO involve vascular manifestations and have similar pathophysiological associations.

The use of the CHA_2_DS_2_-VASc score in ophthalmology is not novel. Pikkel et al. concluded that the CHA_2_DS_2_-VASc score was correlated with glaucoma treatment and prognosis [16]. Furthermore, the relationship between RVO and systemic diseases has already been discussed. Wong et al. showed that RVO and retinal arteriolar emboli are associated with carotid artery disease, hypertension, and other cardiovascular risks [17]. Liu et al. investigated predictive factors for BCVA subsequent to ranibizumab treatment in patients with CME associated with RVO [18]. Their findings suggested that ELM integrity beneath the center of the fovea and baseline BCVA may be more useful than other factors in the prediction of visual function in patients with CME secondary to RVO after receiving ranibizumab injections. Interestingly, Yin et al. examined predictive factors from different studies associated with positive visual activity outcomes in patients with RVO [19]. Younger age, history of hypertension, and male sex were associated with better final RVO.

Here we found that the CHA_2_DS_2_-VASc score of 6 or above was associated with a worse prognosis for visual acuity after RVO. The findings of the study may aid physicians in providing a personal prognosis to each patient who presents with such an event.

This study has several limitations. First, it is possible that several other parameters reported in the literature, were not examined here and may have confounded the results. Few patients received intravitreal steroids in addition to anti-VEGFs. Furthermore, generally patients with a high CHA_2_DS_2_-VASc score, may have had poorer compliance to treatment, since these are with more comorbidities. To control this bias, we included only patients with a 1 year follow-up.

The study's retrospective design limits its ability to establish causality. To confirm these findings and clearly establish a causal relationship between CHA_2_DS_2_-VASc scores and visual acuity outcomes after RVO, prospective studies are needed.

CHADS-VASc scores of six to nine are relatively rare, which poses a challenge in assembling a sufficiently large study cohort. The prevalence of such high scores results in a smaller sample size, which may limit the study's statistical power and the generalizability of its findings. This shortcoming calls for a more extensive data collection effort or multicenter collaboration to ensure robust analysis and reliable conclusions.

We chose to analyze both BRVO and CRVO collectively, given the analogous treatment strategies and clinical protocols applied in such cases. Furthermore, performing subgroup analyses would have further diminished the sample size, potentially compromising the statistical power and reliability of the results.

Single center approach may improve repeatability of the study results by reducing heterogeneity of the study population and clinical practices, on the other hand single center approach may limit the generalizability of the findings.

This is a retrospective study, which can affect the results due to several factors, including variability in the treating physician's assessments, differences in treatment approaches, and inherent biases associated with retrospective research. A multicenter prospective study would mitigate these biases, providing more reliable and consistent data.

In conclusion, with a CHA_2_DS_2_-VASc score of 6 or above, patients with retinal venous occlusion had a poorer prognosis for visual acuity outcomes.

## Figures and Tables

**Table 1:** Baseline variables of the 83 eyes included in this study.

Parameter	
Age	67.9 ± 13.8
Gender (female)	32 (38.6%)
Pseudophakia	26 (32.2%)
BCVA (LogMAR)	0.81 ± 0.67
IOP (mmHg)	16.4 ± 6.7
CHA₂DS₂-VASc score	3.04 ± 1.82
Macular status	
Foveal thickness (*μ*m)	383.3 ± 84.9
CSMT (*μ*m)	470.3 ± 197.3
Macular volume (*μ*m^3^)	10.5 ± 2.4

*Note:* Data are given as mean ± SD or absolute numbers and proportions.

Abbreviations: BCVA, best-corrected visual acuity; CSMT, central subfield macular thickness; IOP, intraocular pressure; LogMAR, logarithm of minimum angle of resolution. CHA_2_DS_2_-VASc stands for congestive heart failure, hypertension, age greater than 75, diabetes, stroke, vascular disease, age between 65 and 75, and sex.

**Table 2:** Baseline parameters and outcomes in regard CHA_2_DS_2_-VASc score.

	CHA_2_DS_2_-VASc score			*p* value
	0–2 (*n* = 31)	3–5 (*n* = 45)	6–9 (*n* = 7)	
Baseline				
Age (years)	59.5 ± 13.7	72.6 ± 11.6	76.0 ± 8.9	<0.001
Gender (female)	5 (16.1%)	24 (53.3%)	3 (42.9%)	0.003
BCVA (LogMAR)	0.64 ± 0.56	0.86 ± 0.67	1.24 ± 0.89	0.068
IOP (mmHg)	14.2 ± 2.7	17.0 ± 6.8	23.0 ± 13.0	0.005
Foveal thickness (*μ*m)	390.7 ± 72.6	381.4 ± 94.4	362.1 ± 74.4	0.714
CSMT (*μ*m)	479.8 ± 182.8	470.4 ± 213.3	427.1 ± 172.7	0.820
Macular volume (*μ*m^3^)	10.8 ± 1.7	10.4 ± 2.87	10.2 ± 2.08	0.780
Pseudophakia eyes	10 (32.25%)	14 (31.11%)	2 (28.57%)	0.812
1-year				
BCVA (LogMAR)	0.52 ± 0.60	0.85 ± 0.85	1.84 ± 1.43	0.001
ΔBCVA (LogMAR)	−0.12 ± 0.59	−0.01 ± 0.65	+0.60 ± 0.94	0.038
IOP (mmHg)	15.7 ± 7.25	15.4 ± 4.8	21.6 ± 10.5	0.060

*Note:* Data are given as mean ± SD or absolute numbers and proportions.

Abbreviations: BCVA, best-corrected visual acuity; CSMT, central subfield macular thickness; IOP, intraocular pressure; LogMAR, logarithm of minimum angle of resolution.

## Data Availability

The raw data used to support the findings of this study can be provided upon request.

## Supporting Information

Supporting Information 1 Bar 1: association between visual acuity and CHADS-VASc score.

Supporting Information 2 Density Plot: VA delta by CHADS-VASc score.

Supporting Information 3 Supporting Figure 1: Flow Diagram of the Inclusion Process.
