# Supplementary figures and images for: CHA2DS2-VASc Score in Predicting Visual Acuity Outcomes Following Retinal Vein Occlusion
Source: J Ophthalmol. 2024 Oct 21;2024:3054783. doi: 10.1155/2024/3054783 (PMC11519066; doi:10.1155/2024/3054783)

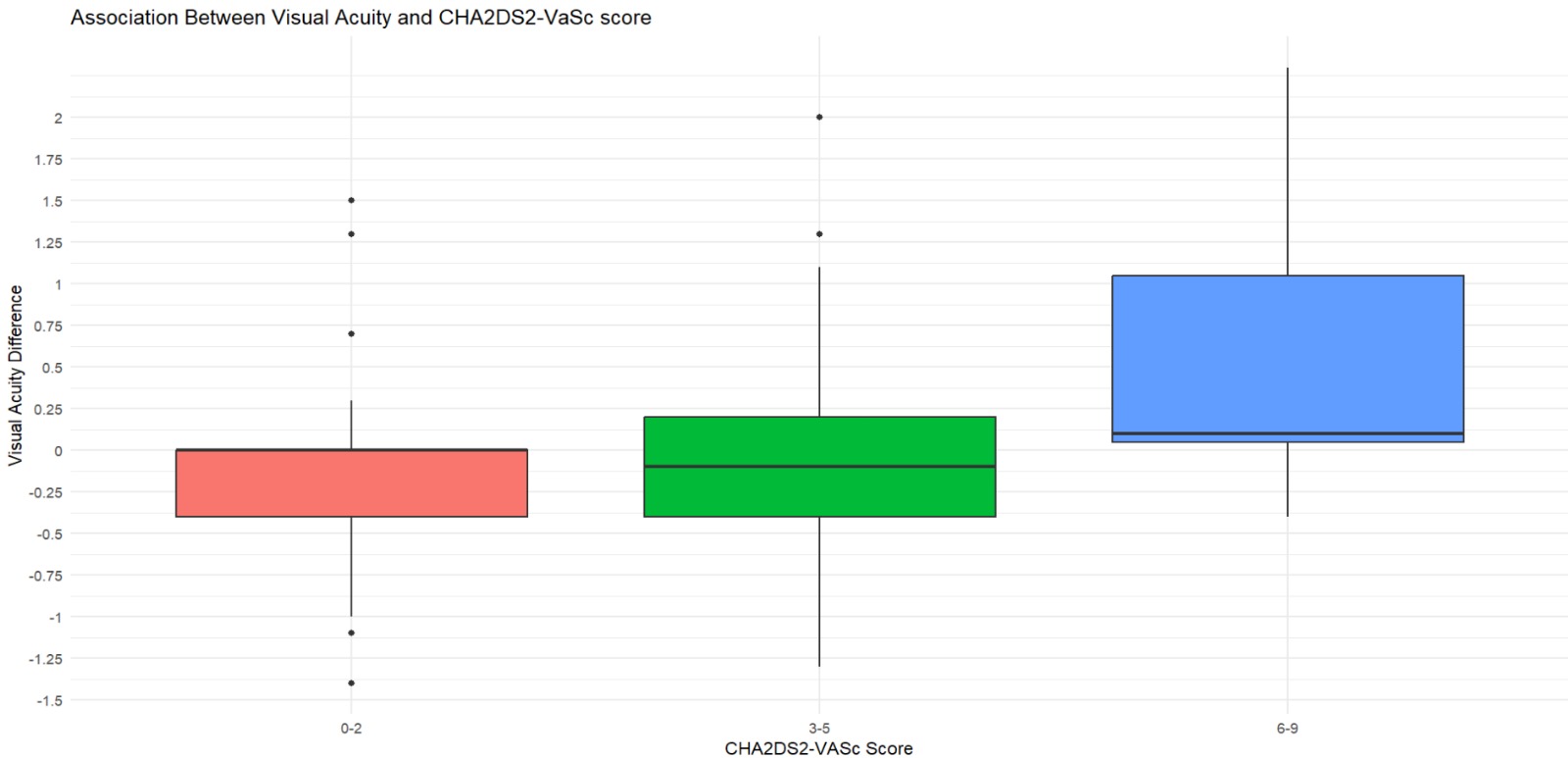

Supplement: Supporting Information 1 — Bar 1: association between visual acuity and CHADS-VASc score. [file 3054783.f1.jpeg]

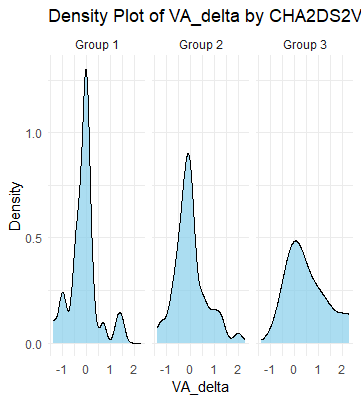

Supplement: Supporting Information 2 — Density Plot: VA delta by CHADS-VASc score. [file 3054783.f2.png]
