# Supplementary material for: CHA2DS2-VASc Score in Predicting Visual Acuity Outcomes Following Retinal Vein Occlusion
Source: J Ophthalmol. 2024 Oct 21;2024:3054783. doi: 10.1155/2024/3054783 (PMC11519066; doi:10.1155/2024/3054783)
Supplement: Supporting Information 3 — Supporting Figure 1: Flow Diagram of the Inclusion Process. [file 3054783.f3.docx]

Supplemental Figure 1. Flow Diagram of the Inclusion Process

Records identified through database searching
(n = 116)

## Screening

## Included

## Eligibility

## Identification

Records after duplicates removed
(n = 110)

Records screened
(n = 110)

Records excluded as irrelevant to our subjects. (total n=18):

Misdiagnosis (n=11), patients who underwent procedures or surgeries during the follow-up period (n=4), patients who did not complete a year of follow-up (n=3)

Records assessed for eligibility
(n = 92)

Records excluded due to incomplete data (n =9)

Records included in final analysis
(n = 83)
